# Elevated serum eotaxin and IP‐10 levels as potential biomarkers for the detection of esophageal squamous cell carcinoma

**DOI:** 10.1002/jcla.23904

**Published:** 2021-07-21

**Authors:** Chen Chang, Min‐Jie Wang, Xiao‐Feng Bi, Zhi‐Yuan Fan, Dan Feng, Hong‐Qing Cai, Yu Zhang, Xin Xu, Yan Cai, Jun Qi, Wen‐Qiang Wei, Jia‐Jie Hao, Ming‐Rong Wang

**Affiliations:** ^1^ State Key Laboratory of Molecular Oncology Center for Cancer Precision Medicine National Cancer Center/National Clinical Research Center for Cancer/Cancer Hospital Chinese Academy of Medical Sciences and Peking Union Medical College Beijing China; ^2^ Department of Clinical Laboratory National Cancer Center/National Clinical Research Center for Cancer/Cancer Hospital Chinese Academy of Medical Sciences and Peking Union Medical College Beijing China; ^3^ Department of Cancer Prevention Office of Cancer Screening National Cancer Center/National Clinical Research Center for Cancer/Cancer Hospital Chinese Academy of Medical Sciences and Peking Union Medical College Beijing China; ^4^ Department of Cancer Epidemiology Center for Cancer Precision Medicine National Cancer Center/National Clinical Research Center for Cancer/Cancer Hospital Chinese Academy of Medical Sciences and Peking Union Medical College Beijing China

**Keywords:** eotaxin, ESCC, esophageal squamous cell carcinoma, IP‐10, serum marker

## Abstract

**Background and Aims:**

Esophageal squamous cell cancer (ESCC) is one of the leading malignant cancers with a high incidence and mortality. Exploring novel serum biomarkers will help improve the management and monitoring of ESCC.

**Methods:**

In the present study, we first used a ProcartaPlex Array to screen for serum proteins that were increased in 40 ESCC patients compared with matched normal controls; we found that eight proteins (IL‐2, IL‐5, IP‐10, IL‐8, eotaxin, TNF‐α, HGF, and MIP‐1b) had higher serum levels in ESCC patients than in normal controls. We further verified the clinical relevance of the candidate biomarkers with a larger sample of sera.

**Results:**

In the 174 tested ESCC patients and 189 normal controls, the serum levels of eotaxin and IP‐10 were significantly higher in patients than in normal controls (*p* = 0.0038, 0.0031). In particular, these two proteins were also elevated in the sera of patients with early‐stage (0‐IIA) ESCC (*p* = 0.0041, 0.0412). When combining CEA and CYFRA21‐1 (in use clinically) with eotaxin or IP‐10, the effectiveness of detecting ESCC was superior to that of CEA and/or CYFRA21‐1 alone. Moreover, the serum level of eotaxin dropped significantly after surgical resection of primary tumors compared with that in preoperative ESCC samples (*p* < 0.001).

**Conclusions:**

The data suggest that serum eotaxin and IP‐10 might be potential biomarkers for the detection of ESCC.

## Graphical abstract

Serum samples from 40 ESCC patients and 40 normal controls were used to screen protein candidates by using ProcartaPlex Array in the biomarker discovery stage. The diagnostic values of the candidate IL‐2, IL‐5, IP‐10, IL‐8, eotaxin, TNF‐α, HGF, MIP‐1b using ELISA were further evaluated in 363 serum samples in the biomarker assessment stage and were finally evaluation value of protein panels for the detection and therapy monitoring of ESCC.

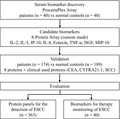

## 1 INTRODUCTION

Esophageal squamous cell cancer (ESCC) is one of the leading malignant cancers in the world. China is one of the regions with the highest incidence rates, and over 90% of cases are esophageal squamous cell carcinomas. Most patients with ESCC have unresectable tumors or radiographically visible metastases at the time of diagnosis, and the overall 5‐year survival rate of this disease has been low.^1^, ^2^ Thus, it is urgent and important to develop new molecular approaches for improving the diagnosis and treatment of ESCC.

Serum proteins are the most commonly used biomarkers in clinical diagnosis and treatment monitoring, with characteristics of being rapid, less invasive, and easy to accept by patients. As reported, α‐fetoprotein (AFP) is the preferred serum marker for the diagnosis and monitoring of hepatocellular carcinoma (HCC).^3^ Cancer testis antigen sperm‐associated antigen 9 (SPAG9) could distinguish lung cancer from normal controls (*p* < 0.001), and the level of the SPAG9 autoantibody in the sera of untreated patients was significantly higher than that in treated patients.^4^ An increase in serum carcinoembryonic antigen (CEA) and carbohydrate antigen 19‐9 (CA19‐9) has been associated with tumor progression and decreased overall survival of colorectal cancer.^5^ Insulin‐like growth factor binding protein 7 (IGFBP7) has been suggested as a serum marker for ESCC with an area under the curve (AUC) of 0.794, and a sensitivity of 40.6% and specificity of 90.7% based on a cutoff of 2.993 ng/ml.^6^ L1 cell adhesion molecule (L1CAM) has diagnostic performance in ESCC with an AUC of 0.644.^7^ CEA, cytokeratin 19 fragment 21‐1 (CYFRA21‐1), and squamous cell carcinoma antigen (SCC) were also evaluated for the diagnosis of ESCC. However, the sensitivity and specificity of these biomarkers are very limited in ESCC.^8^, ^9^

In the present study, we first used a ProcartaPlex Array to screen for serum proteins increased in 40 ESCC patients compared with the normal controls. By further verification of candidate serum markers with a larger sample of sera (174 ESCC patients and 189 normal controls), we found that serum eotaxin and IP‐10 might be potential biomarkers in the diagnosis and therapeutic monitoring of ESCC patients.

## 2 MATERIALS AND METHODS

### 2.1 Study participants

In the biomarker discovery stage, we selected 80 blood samples from ESCC patients and normal controls at the Cancer Hospital, CAMS/PUMC. In the biomarker assessment stage, 363 serum samples, including 174 ESCC samples and 189 normal controls, were collected from the Cancer Hospital, CAMS/PUMC (Table 1). Forty serum samples from ESCC patients before and after surgery were obtained from Linzhou Cancer Hospital. None of the patients had any other disease prior to diagnosis, nor had they received any other treatment prior to surgery. The normal control serum samples were collected from people who underwent physical examination in the hospital. These normal individuals had no history of cancer, diabetes, or cardiovascular disease. They were matched as best as possible to the patient group with respect to age and sex and were eligible for inclusion in the study.

**TABLE 1:** Participant and clinical information

Characteristics	ESCC patients (*n* = 174)	Normal controls (*n* = 189)
Age, years		
Mean ± SD	60.80 ± 8.28	58.94 ± 9.47
Range	38~79	24~78
Gender		
Male	148	136
Female	26	53
Histological grade*		
High (grade 1)	22	
Middle (grade 2)	96	
Low (grade 3)	50	
Tumor location		
Upper	28	
Middle	99	
Lower	47	
Stage		
Ⅰ	28	
Ⅱ	42	
Ⅲ	81	
Ⅳ	23	
Depth of tumor invasion (T staging)		
T1	32	
T2	21	
T3	93	
T4	28	
Regional lymph nodes (N staging)		
N0	69	
N1	74	
N2	22	
N3	9	
Metastasis		
M0	162	
M1	12	

* The histological grade of six are uncertain; SD, standard deviation.

Tumor stage was evaluated after surgery according to the seventh edition of the American Joint Committee on Cancer (AJCC) Cancer Staging Manual,^10^ and tumors with AJCC stages 0‐IIA were defined as early‐stage ESCC in the present study.^11^ This study was approved by the Ethics Committee/Institutional Review Board of the Cancer Institute (Hospital), the Peking Union Medical College, and the Chinese Academy of Medical Sciences (No. 12‐097/631, 16‐084/1163).

### 2.2 Collection of blood samples

Fasting blood samples from cancer patients and normal controls were processed in an identical manner, collected into anticoagulant‐free tubes, and centrifuged at 2500 g for 5 min. The supernatant was then subpacked and frozen at –80°C until the day before the experiment.

### 2.3 Assessment of serum proteins using multiplex immunoassays

Serum samples were detected with ProcartaPlex multiplex immunoassay kits from the eBioscience division of Affymetrix. Briefly, 50 µl of serum was used for cytokine quantification using a MAGPIX® MILLIPLEX® system (Merck) with xPONENT software (Luminex). The data were analyzed with MILLIPLEX® analyst software (Merck) using a cubic spline curve and background subtraction to convert the mean fluorescence intensity to pg/ml values. The kit detected up to 40 protein targets in a single sample.

### 2.4 Measurement of candidate biomarkers by ELISA

ProcartaPlex Mix&Match Human 8‐plex kits (Thermo Fisher) were used to measure the serum levels of the candidate biomarkers IL‐2, IL‐5, IP‐10, IL‐8, eotaxin, TNF‐α, HGF, and MIP‐1b. CEA, CYFRA21‐1, and SCC were simultaneously measured by ELISA‐CEA, ELISA‐CYFRA21‐1 (DRG), and ELISA‐SCC kits (CanAg). All measurements, including samples and standards, were performed in duplicate. The serum concentrations were obtained with a standard curve. ELISA and multiplex immunoassays were performed by different researchers who were blinded to the status of the samples and patients. Samples of patients and normal controls were assayed together in the same batch. Quality control (QC) was carried out as described in previous literature.^12^

### 2.5 Statistical analysis

The obtained data were statistically analyzed with Microsoft Excel, SPSS (version 22.0), and GraphPad Prism 7.0 software. The differences between groups were tested by the Mann–Whitney *U* test. Comparison of positive rates was performed using chi‐squared tests. Receiver operating characteristic (ROC) curves and the area under the ROC curve (AUC) were used to evaluate the predictive performance of candidate biomarkers. *p* < 0.05 was considered statistically significant.

## 3 RESULTS

### 3.1 Biomarker discovery by serum‐based multiplex immunoassays

**FIGURE 1:**
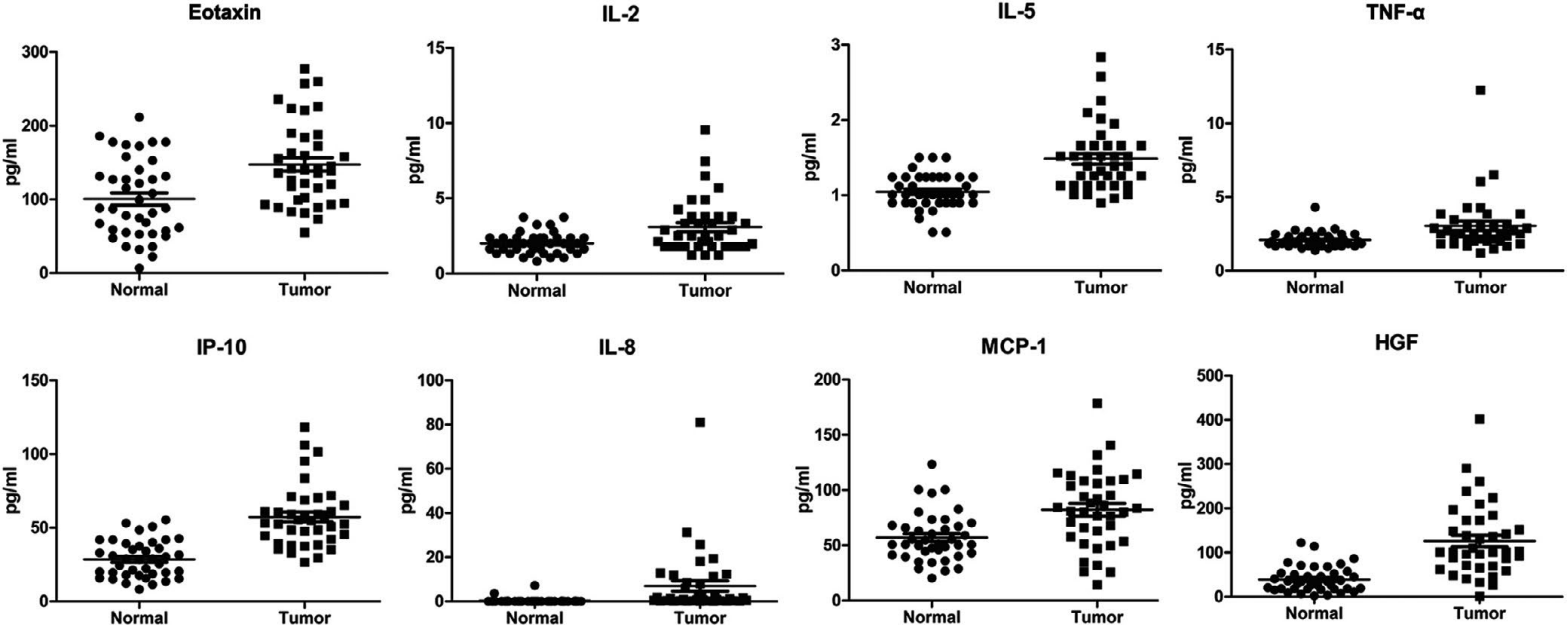
Results of multiplex immunoassay arrays to measure serum proteins in 40 ESCC patients. Differences in serum protein levels between patients and normal controls (*p* < 0.05)

In the biomarker discovery stage, we first analyzed serum samples of 40 ESCC patients and 40 normal controls using ProcartaPlex multiplex kits to initially screen for candidate biomarkers. The results showed that eight proteins (IL‐2, IL‐5, IP‐10, IL‐8, eotaxin, TNF‐α, HGF, and MIP‐1b) had higher serum levels in ESCC patients than in normal controls (Figure 1).

### 3.2 Serum levels of candidate biomarkers in ESCC patients and normal controls

**FIGURE 2:**
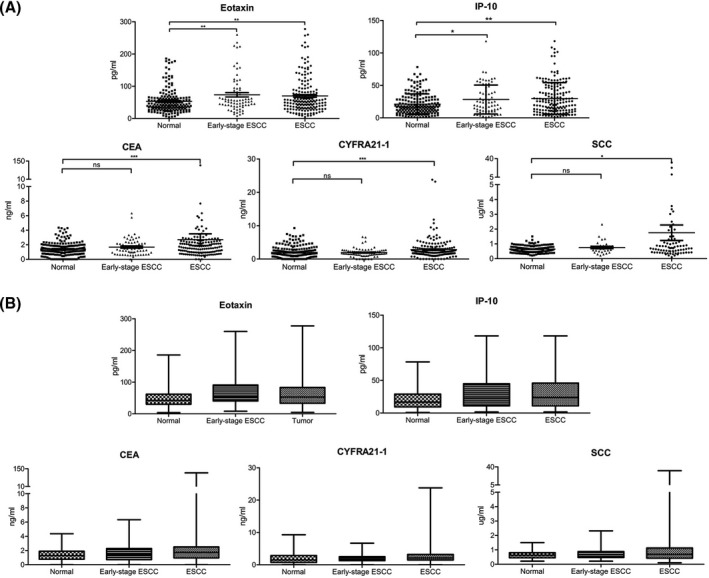
The serum levels of candidate proteins in 174 ESCC patients and 189 healthy controls. (A) The concentrations of the serum biomarkers are shown in scatter plots. The center line represents the mean. (B) Box plot showing the degree of dispersion. The center line represents the median. (*p < 0.05, **p < 0.01, ***p < 0.001)

We then expanded the serum sampling to 174 ESCC patients and 189 normal controls to verify the clinical value of the eight candidate biomarkers, IL‐2, IL‐5, IP‐10, IL‐8, eotaxin, TNF‐α, HGF, and MIP‐1b. The results showed that among the above candidate biomarkers, eotaxin was the most effective for detecting ESCC. Serum levels of eotaxin and IP‐10 were significantly higher in patients than in controls (*p* = 0.0038, 0.0031) (Figure 2; Figure S1). In particular, elevated serum eotaxin and IP‐10 were also observed in patients with early‐stage (0‐IIA) tumors (*p* = 0.0041, 0.0412; Table 2). In addition, high eotaxin was correlated with N staging (*p* = 0.042). However, there was no statistically significant association between the other six serum proteins and clinicopathological parameters (all *p* > 0.05) (Table 3).

**TABLE 2:** Differences in serum protein levels between patients with ESCC or early‐stage ESCC and normal controls

Protein	Normal controls	ESCC	Early‐stage ESCC
CEA			
*N*	180	145	62
Mean ± SD	1.41 ± 0.93	2.70 ± 9.68	1.68 ± 1.18
*p* Value		0.0002	0.2296
CYFRA21‐1			
*N*	189	174	73
Mean ± SD	1.99 ± 1.86	2.84 ± 2.99	1.95 ± 1.28
*p* Value		<0.0001	0.2096
SCC			
*N*	131	76	29
Mean ± SD	0.63 ± 0.25	1.75 ± 4.56	0.76 ± 0.43
*p* Value		0.0478	0.3781
IP‐10			
*N*	189	174	73
Mean ± SD	21.04 ± 15.85	29.80 ± 24.47	28.25 ± 22.33
*p* Value		0.0031	0.0412
Eotaxin			
*N*	189	174	73
Mean ± SD	52.91 ± 36.55	69.82 ± 54.67	73.44 ± 57.10
*p* Value		0.0038	0.0041
HGF			
*N*	189	174	73
Mean ± SD	204.1 ± 192.3	249.8 ± 192.3	218.0 ± 163.4
*p* Value		0.0779	0.3462
MIP‐1b			
*N*	189	174	73
Mean ± SD	70.97 ± 85.55	55.32 ± 58.95	53.03 ± 67.70
*p* Value		0.4445	0.1608
IL‐2			
*N*	189	174	73
Mean ± SD	2.490 ± 1.111	2.452 ± 1.396	2.241 ± 0.9539
*p* Value		0.0241	0.0058
IL‐5			
*N*	189	174	73
Mean ± SD	3.723 ± 11.89	2.052 ± 1.167	1.968 ± 1.210
*p* Value		0.9254	0.5599
IL‐8			
*N*	189	174	73
Mean ± SD	4.339 ± 9.870	4.554 ± 11.61	3.292 ± 9.947
*p* Value		0.0805	0.0202
TNF‐α			
*N*	189	174	73
Mean ± SD	2.551 ± 2.876	2.218 ± 1.138	2.020 ± 0.6827
*p* Value		0.0421	0.0054

* Comparison of serum protein levels between patients with ESCC or early‐stage ESCC and normal controls; early‐stage: stages 0‐IIA; SD, standard deviation.

**TABLE 3:** Correlation between biomarkers and clinicopathological characteristics of ESCC patients

	CEA (%)	CYFRA21‐1 (%)	SCC (%)	Eotaxin (%)	IP‐10 (%)
Age at surgery, years					
≤60	29 (43.9)	78 (82.1)	10 (32.3)	29 (36.7)	23 (29.11)
>60	33 (41.8)	65 (82.3)	13 (28.9)	31 (32.6)	30 (31.58)
*p* Value	0.793	0.6	0.753	0.573	0.725
Gender					
Male	55 (46.2)	120 (81.1)	21 (34.4)	48 (32.4)	43 (29.05)
Female	7 (26.9)	23 (88.5)	2 (13.3)	12 (46.2)	10 (38.46)
*p* Value	0.072	0.529	0.201	0.175	0.677
Histology grade					
G1	11 (55.0)	19 (86.4)	3 (33.3)	8 (36.4)	5 (22.73)
G2	33 (41.8)	80 (83.3)	13 (31.0)	33 (34.4)	31 (32.29)
G3	16 (39.0)	39 (78.0)	5 (23.8)	18 (36.0)	15 (30.00)
*p* Value	0.475	0.623	0.806	0.907	0.677
Tumor location					
Upper	9 (36.0)	22 (78.6)	3 (27.3)	8 (28.6)	9 (32.14)
Middle	40 (47.1)	80 (80.8)	12 (26.7)	41 (41.4)	32 (32.32)
Lower	13 (37.1)	41 (87.2)	8 (40.0)	11 (23.4)	12 (25.53)
*p* Value	0.458	0.55	0.543	0.078	0.691
Depth of tumor invasion (T staging)					
T1+T2	16 (36.4)	42 (79.2)	4 (16.7)	15 (39.5)	14 (26.42)
T3+T4	46 (45.5)	101 (83.5)	19 (36.5)	45 (37.2)	39 (32.23)
*p* Value	0.304	0.503	0.138	0.256	0.443
Regional lymph nodes (N staging)					
N0	29 (37.2)	73 (80.2)	11 (26.2)	25 (27.5)	25 (27.47)
N1+N2+N3	33 (49.3)	70 (84.3)	12 (37.5)	35 (42.2)	28 (33.73)
*p* Value	0.143	0.478	0.298	0.042	0.37
Stage					
Stages 0‐ⅡA	22 (35.5)	58 (79.5)	5 (17.2)	24 (32.9)	22 (30.14)
Stage ⅡB‐Ⅳ	40 (48.2)	85 (84.2)	18 (38.3)	36 (35.6)	31 (30.69)
*p* Value	0.126	0.423	0.052	0.705	0.937

We simultaneously measured the classical tumor biomarkers CEA, CYFRA21‐1, and SCC and found that serum CEA, CFYRA21‐1, and SCC levels were also higher in ESCC patients than in healthy controls (Figure 2).

### 3.3 Performance of serum biomarkers for detecting advanced and early‐stage ESCC

**FIGURE 3:**
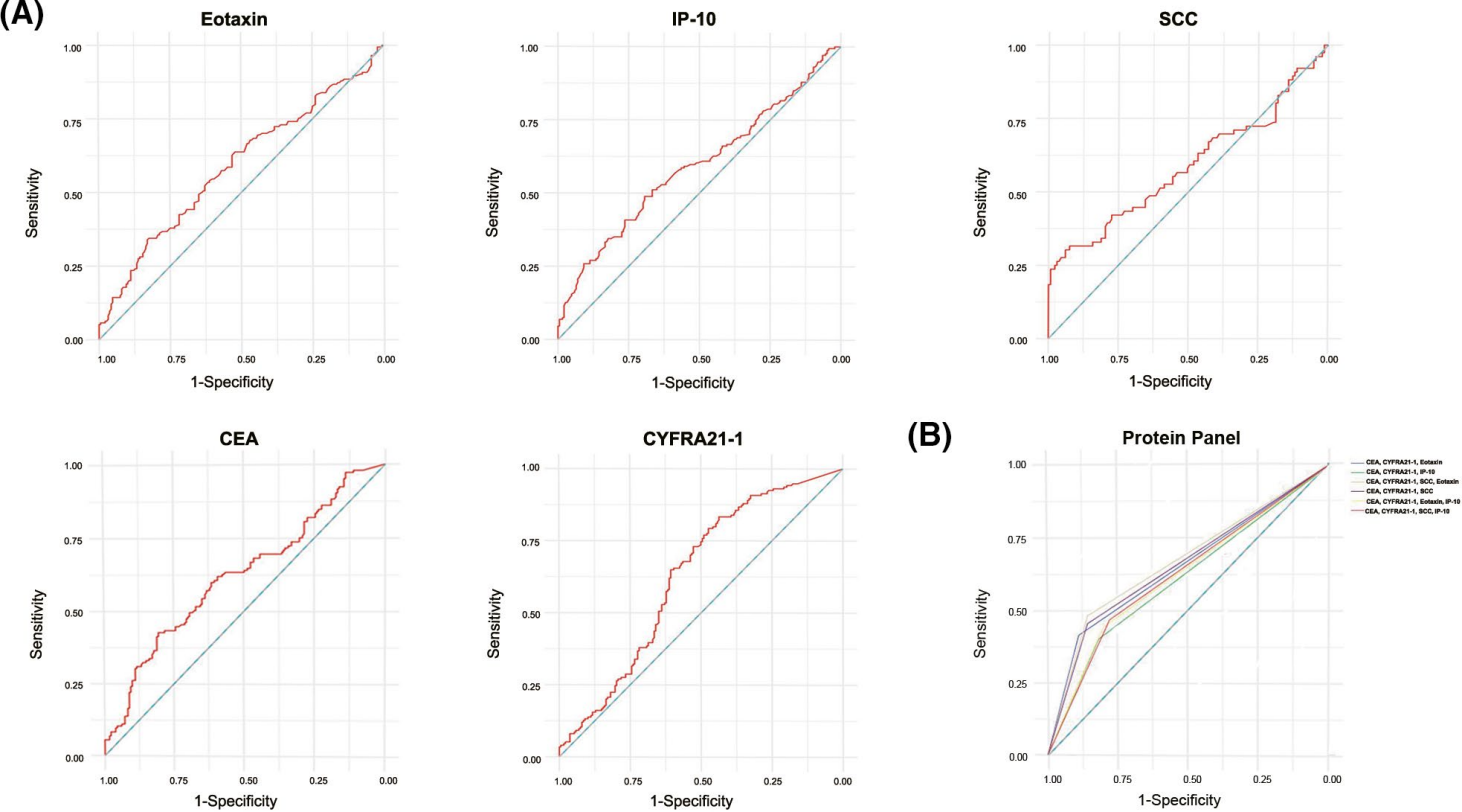
ROC curves of serum biomarkers for detecting ESCC. The area under the green line is 0.5

We further analyzed the ROC curves of candidate serum biomarkers to assess their diagnostic value (Figure 3A). We found that serum levels of eotaxin, IP‐10, CEA, CYFRA21‐1, and SCC could differentiate ESCC patients from healthy controls, with AUCs of 0.588, 0.590, 0.619, 0.626, and 0.589, respectively. In accordance with the ROC curve derived from the ESCC group versus the control group, the optimal cutoff value of eotaxin was 68.23 pg/ml (sensitivity 34.50%, specificity 82.44%) and that of IP‐10 was 37.70 pg/ml (sensitivity 30.05%, specificity 84.90%). For CEA, CYFRA21‐1, and SCC, the optimal cutoff values were 2.01 ng/ml (sensitivity 42.80%, specificity 80.56%), 1.22 ng/ml (sensitivity 83.30%, specificity 43.39%), and 1.02 µg/ml (sensitivity 30.30%, specificity 93.98%), respectively (Table 4). In patients with early‐stage tumors, the sensitivity of eotaxin was 32.88% (Table 5).

**TABLE 4:** Evaluation of serum protein biomarkers for detecting ESCC

Protein	AUC	Cutoff value	95% CI	Sensitivity	Specificity	Accuracy
CEA	0.619	2.01 ng/ml	0.558–0.681	42.76%	80.56%	63.69%
CYFRA21‐1	0.626	1.22 ng/ml	0.568–0.684	83.33%	43.39%	62.53%
SCC	0.589	1.02 µg/ml	0.503–0.675	30.26%	93.98%	70.81%
Eotaxin	0.588	68.23 pg/ml	0.529–0.647	34.48%	82.45%	59.39%
IP−10	0.59	37.70 pg/ml	0.531–0.649	30.05%	84.90%	58.01%

Abbreviations: AUC, area under the curve; CI, confidence interval.

**TABLE 5:** Evaluation of serum protein biomarkers for detecting early‐stage ESCC

Protein	AUC	Cutoff value	95% CI	Sensitivity	Specificity	Accuracy
CEA	0.551	2.01 ng/ml	0.465–0.637	35.48%	80.56%	69.01%
CYFRA21‐1	0.550	1.22 ng/ml	0.479–0.621	79.45%	43.39%	53.44%
SCC	0.559	1.02 µg/ml	0.437–0.682	17.24%	93.98%	80.25%
Eotaxin	0.615	68.23 pg/ml	0.538–0.692	32.88%	82.45%	68.58%
IP−10	0.582	37.70 pg/ml	0.500–0.663	30.14%	84.90%	68.70%

Abbreviations: AUC, area under the curve; CI, confidence interval.

### 3.4 Sensitivity and specificity of serum protein panels in detecting ESCC

We further analyzed the combinations of different serum proteins and found that eotaxin and/or IP‐10 combined with CEA and CYFRA21‐1 were superior to CEA and/or CYFRA21‐1 alone. When two out of the combinations were positive, the accuracies of eotaxin/CEA/CYFRA21‐1, IP‐10/CEA/CYFRA21‐1, and eotaxin/IP‐10/CEA/CYFRA21‐1 were 72.22%, 70.19%, and 69.47%, and the sensitivities were 61.38%, 59.31%, and 65.52%, respectively. The AUC for the combination of the proteins was higher than that for each single protein (Figure 3B; Table 6). In particular, the three combinations also showed higher sensitivity and/or accuracies for patients with early‐stage (0‐IIA) ESCC (Table 7).

**TABLE 6:** The sensitivities, specificities, and accuracies of protein panels for detecting ESCC

Protein panel	AUC	Sensitivity	Specificity	Accuracy
CEA, CYFRA21‐1, Eotaxin	0.650	61.38%	81.01%	72.22%
CEA, CYFRA21‐1, IP‐10	0.609	59.31%	79.10%	70.19%
CEA, CYFRA21‐1, SCC, Eotaxin	0.668	47.37%	86.47%	72.25%
CEA, CYFRA21‐1, SCC	0.654	44.74%	86.47%	71.29%
CEA, CYFRA21‐1, Eotaxin, IP‐10	0.621	65.52%	72.73%	69.47%
CEA, CYFRA21‐1, SCC, IP‐10	0.623	46.05%	78.46%	66.50%

Abbreviation: AUC, area under the curve.

**TABLE 7:** The sensitivities, specificities, and accuracies of protein panels for detecting early‐stage ESCC

Protein panel	AUC	Sensitivity	Specificity	Accuracy
CEA, CYFRA21‐1, Eotaxin	0.650	56.45%	81.01%	74.69%
CEA, CYFRA21‐1, IP‐10	0.646	53.23%	79.10%	72.38%
CEA, CYFRA21‐1, SCC, Eotaxin	0.646	31.03%	86.47%	76.54%
CEA, CYFRA21‐1, SCC	0.575	24.14%	86.47%	75.31%
CEA, CYFRA21‐1, Eotaxin, IP‐10	0.642	59.68%	72.73%	69.33%
CEA, CYFRA21‐1, SCC, IP‐10	0.633	24.14%	78.46%	68.55%

Abbreviation: AUC, area under the curve.

### 3.5 Difference in serum eotaxin levels between preoperative and postoperative patients

**FIGURE 4:**
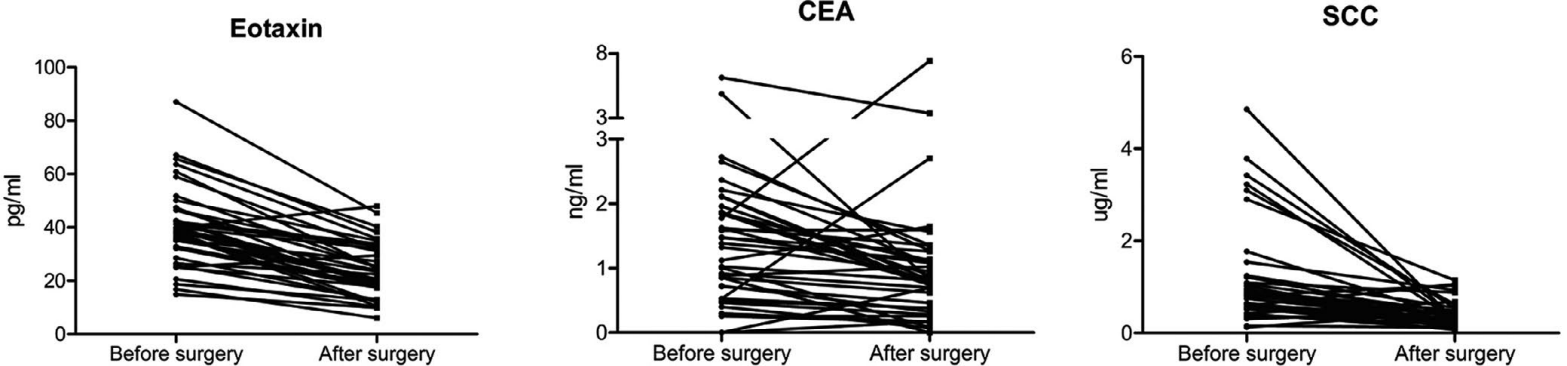
Changes in serum biomarker levels before and after surgical resection of ESCC

We carried out a comparative analysis of serum samples from 40 ESCC patients before and after surgery, in which postoperative samples were taken at 1 week after tumor resection. The results showed that the level of serum eotaxin in postoperative samples decreased significantly compared with that preoperatively (*p* < 0.0001). A similar postoperative decrease was also observed for CEA and SCC (Figure 4; Figure S2).

## 4 DISCUSSION

Identifying new and more effective biomarkers has always been a clinical challenge for the management of ESCC. In the present study, we found that serum eotaxin and IP‐10 are good potential candidates. Several studies have demonstrated that elevated eotaxin levels are of diagnostic or prognostic value for cancers such as prostate cancer, renal cell cancer, gastric cancer, colorectal cancer, and ovarian cancer.^13^, ^14^, ^15^, ^16^, ^17^ Other investigations have shown the implication of IP‐10 in cervical carcinoma^18^ and breast cancer.^19^ However, there is no information available on the association of eotaxin or IP‐10 with ESCC.

In this study, we observed that serum eotaxin has diagnostic value in ESCC with an AUC of 0.588, a specificity of 82.44%, and a sensitivity of 34.50%, and IP‐10 has an AUC of 0.590, a specificity of 84.9%, and a sensitivity of 30.05%. In particular, these two proteins also had elevated serum levels in patients with early‐stage (0‐IIA) ESCC. Furthermore, eotaxin levels were positively correlated with tumor N stage. The combination of several biomarkers is a common strategy to improve diagnostic sensitivity and specificity in studies of biomarkers. We found that the AUC of serum eotaxin and/or IP‐10 combined with CEA and CYFRA21‐1 was higher than that of CEA and/or CYFRA21‐1 alone, including for patients with both advanced and early‐stage (0‐IIA) ESCC. Serum marker detection is generally used as an important auxiliary pre‐examination method. For esophageal cancer, if a certain marker that may be associated with this disease is clinically found to be elevated in the serum of a patient, endoscopy scans and tissue biopsy are immediately performed (if possible) for further examination to confirm the diagnosis. Based on such clinical practice, when two or several markers (or panels) are close or similar in both accuracy and specificity, those with higher sensitivity should be selected to minimize the rate of missed diagnosis upon serological examination. In the present work, we found that three panels of biomarkers eotaxin/CEA/CYFRA21‐1, IP‐10/CEA/CYFRA21‐1, and eotaxin/IP‐10/CEA/CYFRA21‐1 have high clinical potential. Compared with classical CEA, CYFRA21‐1, or SCC alone, none of the three panels showed a reduced diagnostic efficiency (AUC and accuracy), and the sensitivities (59.31%–65.52%) of all three panels were much higher than those of classical CEA or SCC alone (42.76% and 30.26%). In addition, using the same cutoff values, the specificity of CYFRA21‐1 alone (43.39%) is much lower than the panels (72.23%–81.01%), despite the fact that CYFRA21‐1 has a higher sensitivity when used alone compared to the panels. Taken together, the data in the present manuscript demonstrate that the three biomarker panels (eotaxin/CEA/CYFRA21‐1, IP‐10/CEA/CYFRA21‐1, and eotaxin/IP‐10/CEA/CYFRA21‐1) are significantly superior to the single markers commonly used in the clinic.

Interferon‐γ‐induced protein 10 kDa (IP‐10), also known as C‐X‐C motif chemokine 10 (CXCL10) or small‐inducible cytokine B10, is a cytokine belonging to the CXC chemokine subfamily that binds to the common receptor CXCR3. IP‐10 can be produced by various types of cells in response to IFN‐γ.^20^ Alterations in IP‐10 expression levels have been associated with inflammatory diseases, including infectious diseases, immune dysfunction, and tumor development. IP‐10 is also recognized as a biomarker that predicts the severity of various diseases.^21^ IP‐10 is a pleiotropic molecule capable of exerting potent biological functions, including promoting the chemotactic activity of CXCR3‐positive cells, inducing apoptosis, and regulating cell growth and proliferation as well as angiogenesis in infectious and inflammatory diseases and cancer.^22^, ^23^, ^24^ IP‐10 has strong anti‐condyloma acuminatum effects, inducing apoptosis and inhibiting HPV in cervical carcinoma.^18^ In addition, IP‐10 is a major natural killer (NK) cell recruiting chemokine, and it exerts antitumor effects by inhibiting angiogenesis,^25^ making IP‐10 a potent antitumor factor.^26^ Regarding cancer studies, overexpression of IP‐10 in human cancer is mediated through the Raf, PI3K, p38/MAPK, JNK/MAPK, and NF‐kB signaling cascades, which promote cell proliferation and contribute to the development of tumors.^27^, ^28^ Moreover, IP‐10 induces antitumor and antimetastatic activities in different ways, including through immunological and antiangiogenic mechanisms.^29^ Therefore, IP‐10 immunotherapy is considered a promising strategy for breast cancer treatment.^19^ In the present study, we found that serum levels of IP‐10 in ESCC patients were significantly higher than those in normal controls. In particular, this protein was also elevated in the sera of patients with early‐stage (0‐IIA) ESCC, suggesting that serum IP‐10 is a potential early biomarker of the disease.

Eotaxins are C‐C motif chemokines that were first identified as potent eosinophil chemoattractants. Chemokines are responsible for promoting leukocyte attraction to sites of inflammation and cancer. Additionally, some chemokines may promote and regulate metastasis and angiogenesis.^30^, ^31^ Chemokines are a family of secreted proteins that act through autocrine or paracrine mechanisms, and they are thought to influence tumor development.^32^ The eotaxin family currently includes three members: eotaxin‐1 (CCL11), eotaxin‐2 (CCL24), and eotaxin‐3 (CCL26).^33^ Eotaxin‐1 is customarily referred to simply as eotaxin and plays a central role in eosinophil trafficking and is mediated by CC chemokine receptor‐3 (CCR‐3), a seven‐transmembrane‐domain G‐protein‐coupled chemokine receptor that has the highest affinity for CCL11.^34^, ^35^ As mentioned above, eotaxin has been reported to be associated with a variety of human cancers, including the present observation in ESCC. Increasing evidence has demonstrated that eotaxin facilitates the proliferation, migration, and invasion of cancer cells,^16^, ^35^, ^36^, ^37^ which supports the clinical implication of eotaxin being present in patients with ESCC.

Notably, eotaxin serum levels are significantly decreased after tumor resection in ESCC patients, suggesting that this serum protein might have potential in the postoperative surveillance of ESCC patients. To further explore whether serum eotaxin could be a useful biomarker for therapeutic monitoring, larger sample sizes and long‐term follow‐up studies of ESCC patients that undergo surgical treatment are necessary.

## 5 CONCLUSION

In summary, we evaluated the clinical relevance of serum eotaxin and IP‐10 for detecting ESCC for the first time. Our data demonstrate that serum eotaxin and IP‐10 are potential biomarkers for early diagnosis and that eotaxin can also be used to monitor treatment progress.

## CONFLICT OF INTEREST

The authors declare that this research is not related to any commercial or financial interests.

## AUTHOR CONTRIBUTIONS

Chen Chang, Jia‐Jie Hao, and Ming‐Rong Wang conceived and designed the experiments, as well as wrote the paper; Min‐Jie Wang, Xiao‐Feng Bi, Zhi‐Yuan Fang, Dan Feng, Hong‐Qing Cai, Jun Qi, and Wen‐Qiang Wei enrolled the samples and collected the clinical data. Chen Chang performed the experiments and analyzed the data. Jia‐Jie Hao and Yu Zhang interpreted the data. Xin Xu and Yan Cai provided necessary materials. Ming‐Rong Wang and Jia‐Jie Hao jointly supervised the research.

## Supporting information

Fig S1

Fig S2

## Data Availability

The datasets used and/or analyzed during the current study are available from the corresponding author on reasonable request.
