# Supplementary figures and images for: Elevated serum eotaxin and IP‐10 levels as potential biomarkers for the detection of esophageal squamous cell carcinoma
Source: J Clin Lab Anal. 2021 Jul 21;35(9):e23904. doi: 10.1002/jcla.23904 (PMC8418505; doi:10.1002/jcla.23904)

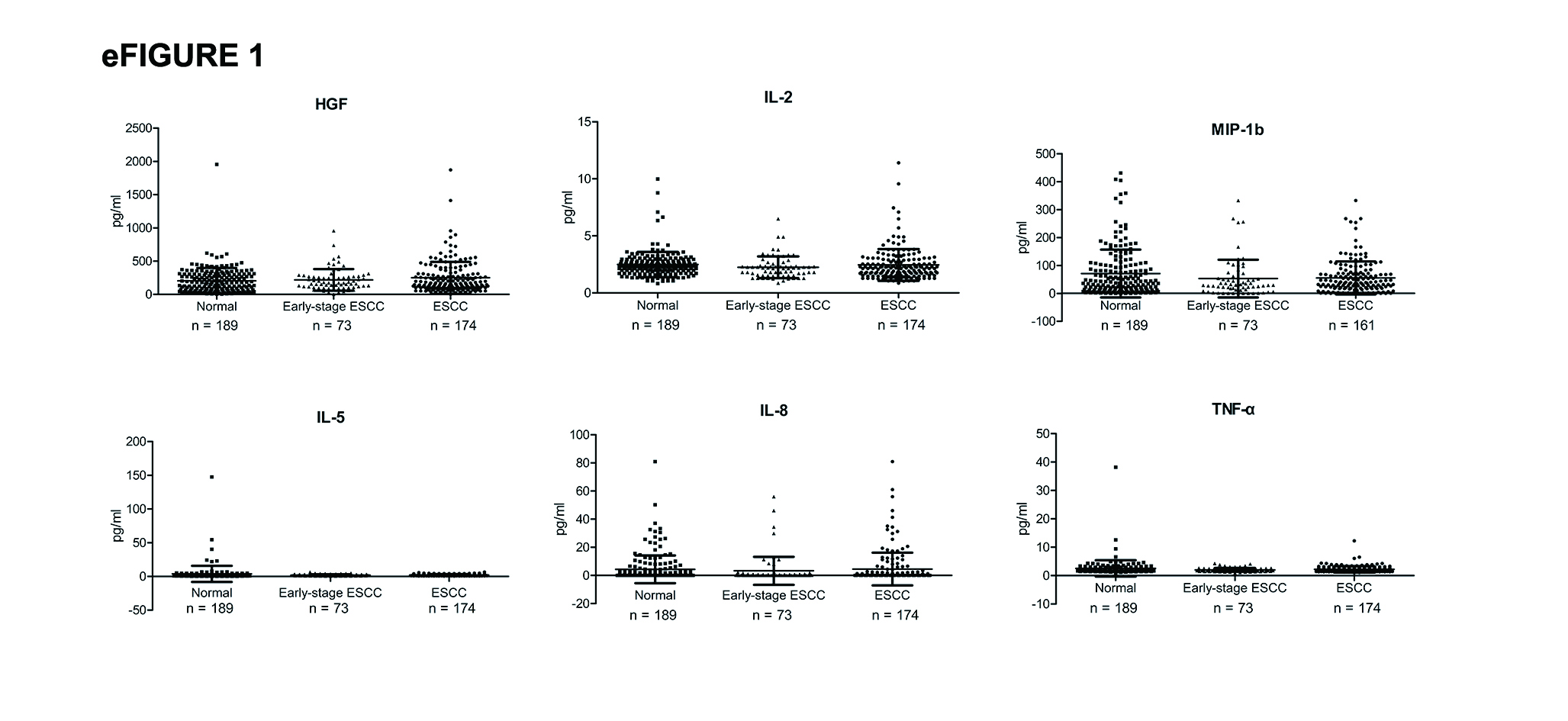

Supplement: Supplementary file 1 — Fig S1 [file JCLA-35-e23904-s002.jpg]

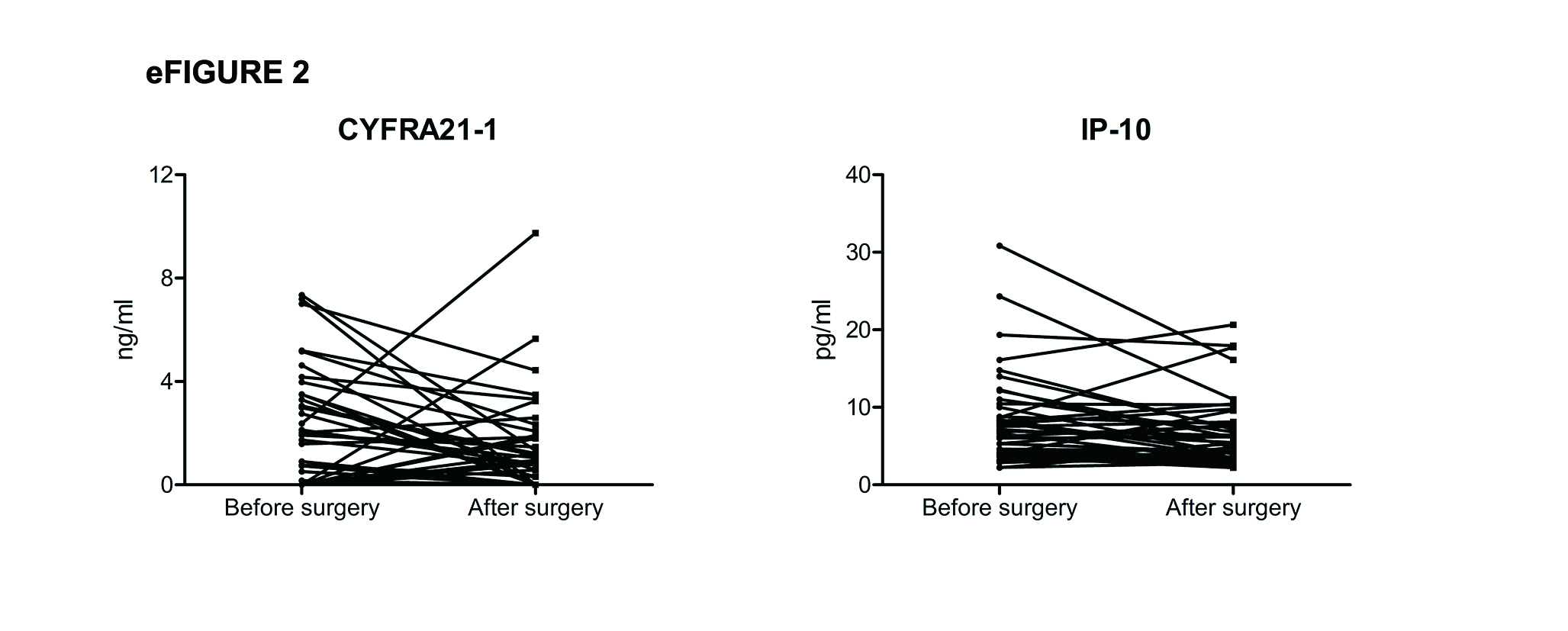

Supplement: Supplementary file 2 — Fig S2 [file JCLA-35-e23904-s001.jpg]
